# Treatment Complications of Head and Neck Cancers and Rehabilitation Measures: A Narrative Review

**DOI:** 10.7759/cureus.61173

**Published:** 2024-05-27

**Authors:** Swati Sharma, Amitabh Kumar Upadhyay, Aaditya Prakash, Pankaj Singodia, Sarat Ravi Kiran, Rama Shankar

**Affiliations:** 1 Prosthodontics, Crown and Bridge & Oral Implantology, Tata Main Hospital, Jamshedpur, IND; 2 Medical Oncology, Tata Main Hospital, Jamshedpur, IND; 3 Radiation Oncology, Tata Main Hospital, Jamshedpur, IND; 4 Plastic Surgery, Tata Main Hospital, Jamshedpur, IND; 5 Oral and Maxillofacial Surgery, Tata Main Hospital, Jamshedpur, IND

**Keywords:** treatment, complications, oral, head and neck cancer, rehabilitation

## Abstract

Head and neck cancers (HNCs) are malignant tumors mainly from squamous cells in the head and neck tissues. Treatment involves a multidisciplinary approach with surgery, radiotherapy, and chemotherapy. However, the long-term prognosis for patients with advanced-stage tumors is guarded, with a median survival time of approximately 24 months. HNC patients have very high rates of depression and anxiety and the highest suicide rate among all cancers due to the intense and challenging nature of the treatment, underscoring the importance of our collective efforts. Rehabilitation success depends on various factors, including tumor, patient, and treatment-related factors. Patients may require post-treatment oral rehabilitation measures, including implants, obturators, and flexible dentures. These measures are crucial, but they often need to be more utilized. Patients may face challenges in maintaining oral hygiene and managing mucositis. Additionally, it is essential to address other intricacies such as trismus, xerostomia, gustatory dysfunctions, neuropathy, speech impairments, and psychological disturbances. Unfortunately, there is little literature on post-treatment rehabilitative measures. Despite its crucial role in improving patients' quality of life, rehabilitation often receives inadequate attention compared to treatment. Our narrative review, which covers various factors that affect rehabilitation, including oral rehabilitation measures and post-treatment complications, is anticipated to deliver practical insights to professionals and inspire positive changes in their regular practice.

## Introduction and background

Epidemiology

Head and neck cancers (HNCs) are malignant tumors mainly originating from squamous cells (HNSCC) in the head and neck tissues. These tumors can arise from various locations, such as the oral cavity, hypopharynx, nasopharynx, oropharynx, lip, nasal cavity, paranasal sinuses, and salivary glands [1]. HNC is a considerable health problem that impacts individuals all over the globe. As per the GLOBOCAN 2020, HNC is the seventh most common cancer globally, accounting for 4.5% of all cancer diagnoses, or 890,000 new cases annually [1,2]. Unfortunately, HNC also causes around 450,000 deaths per year, accounting for about 4.6% of global cancer deaths [1,2]. The incidence rates of HNCs are about 380,000 for the lip and oral cavity, 185,000 for the larynx, 133,000 for the nasopharynx, 98,000 for the oropharynx, 84,000 for the hypopharynx, and 54,000 for the salivary glands [1,2]. HNSCC has varying incidence and mortality rates depending on geographic location and demographics. It is more frequently diagnosed in men than women, with a male-to-female ratio of about 2:1, and in individuals over 50 years of age. HNSCC has the highest incidence rates in South and Southeast Asia due to the prevalent consumption of carcinogenic areca nut [3,4]. India has the highest incidence of HNSCC, with up to 80% of cases related to tobacco use [3-5]. The incidence of HNSCC is increasing worldwide; by 2030, it is projected to increase by 30% each year [3].

Treatment approach

The therapy of advanced oral cancer mandates a multidisciplinary involvement with ablative surgery, frequently with reconstructive surgery, followed by radiotherapy and chemotherapy in a variety of combinations [6]. The treatment of HNC has evolved gradually in the 19th and 20th centuries. In the early 19th century, cervical dissection was proposed for the surgical management of oral cancers. In 1963, conservative cervical dissection was developed to preserve the accessory nerves [7]. In addition, the development of reconstructive surgery has also contributed significantly to improving patients' quality of life (QoL), and robotic surgeries are becoming more common [8]. Surgery is the preferred modality for oral cancer treatment in both the elderly and the young [8]. Linear accelerator radiation therapy, like intensity-modulated radiation therapy (IMRT) and chemotherapy, is used as adjuvant therapy for advanced cancer [6,9]. If cancers are unresectable, then a definitive dose of chemo-radiation therapy is used. Palliative chemotherapy, immunotherapy, and targeted therapies are used in unresectable, recurrent, and metastatic cases [9]. Despite all the coordinated extensive treatments offered, the long-term prognosis for patients with advanced-stage tumors is guarded. The median survival time of patients with oral cancer is approximately 24 months, of which more than six months are consumed in the implementation and completion of the required treatment for primary cancer [10]. 

Treatment can result in several deformities, including esthetic deformity, functional deformity due to loss of continuity of the mandibular arch, and loss of dentition. The optimal recuperation process after advanced cancer treatment involves various elements such as restoring the patient's external appearance, revamping the mandibular arch and facial silhouette, maintaining or recovering oral competence, reviving clear speech, attaining stable dentition to allow chewing of all types of food, and maintaining or reviving the ability to swallow [10]. The primary objective of treating oral cancer is to control it long-term and restore all oral functions for an optimal QoL. However, achieving meaningful long-term oral rehabilitation may be possible only for a few patients. It is challenging to achieve the ability to articulate clear speech, chew everyday foods, and swallow all types of foods [10]. Additionally, sensation to the teeth anterior to the site of mandible resection can also be lost. Surgical reconstruction of the mandible often results in an acceptable external appearance but minor restoration of oral function [10].

With an appropriate team approach to oral health management and rehabilitation, patients who have undergone intense oral cancer treatment can return to society. The suicide rate for oral cancer patients is the highest among all cancers, reflecting the intense and challenging nature of oral cancer treatment [11]. These issues are not only challenging for patients and their families but also for healthcare providers. A review article by Licitra et al. stated that the core team for oral cancer requires intervention from 20 professionals [12]. The extended team requires intervention from 32 professions, including oral management specialists such as oral and maxillofacial surgeons, dentists, and dental hygienists [12]. There are very few studies on oral health management as a concept that encompasses oral function management, oral hygiene management, and oral care in oral cancer treatment [13]. In addition, there are no established follow-up programs for post-treatment oral cancer patients in daily clinical practice. Thus, in advanced staged patients, preoperative treatment planning, inclusive of the maxillofacial prosthodontics team, reconstructive surgery team, and ablative surgeons, should take place to devise a plan of resection, reconstruction, and rehabilitation in order to achieve the desired outcome. 

Objective

The objective of the study is to make professionals aware of the various rehabilitation measures that are underutilized in real-world clinical practice and utilize them in their regular practice. The article provides a narrative review focusing on oral health management from a multidisciplinary and supportive care perspective in oral cancer treatment. 

## Review

Factors affecting rehabilitation

The feasibility of comprehensive rehabilitation of all oral functions depends on many aspects described here.

Tumor Factors

Early-stage primary malignant tumors (T1 and T2) of the oral cavity and some parts of the oropharynx can be addressed efficiently with simple surgical resection without significant reconstructive endeavor or adjuvant treatment. In contrast, advanced cancers mandate major ablative and reconstructive surgery and adjuvant treatment with radiation or chemo-radiation [14,15]. Advanced tumors involving the mandible or adjoining mandible mandate either marginal or segmental mandibulectomy with loss of teeth and teeth-bearing bone [14]. 

Patient Factors

Geriatric patients who are edentulous are not ideal candidates for any noteworthy dental rehabilitation following ablative surgery due to severely atrophic mandible. The mandible usually lacks adequate residual bone for holding endosseous implants for fixed dentures and lacks sufficient alveolar ridge or sulci to support traditional removable dentures [15,16]. Ultimately, these patients will experience suboptimal oral function, leading to a lower QoL. A significant number of patients have restricted food intake and are only able to consume pureed foods via their mouth. Due to comorbidities, these patients are usually not fit for major surgical procedures as well. Lifestyle factors such as oral tobacco and alcohol consumption, along with poor dental hygiene, create an unsuitable environment for effective dental restoration [17,18].

Treatment Factors 

A patient's appearance and oral functions, such as speech clarity, mastication, and swallowing, can be heavily impacted by treatment modalities. Retention or rehabilitation of oral competence and dentition are essential to restoring oral function. 

Surgery: Surgical treatment of oral cancer can lead to extensive changes in the mouth, including loss of mucosal surface, soft tissue volume, effacement of the vestibular sulci, and bone loss if any part of the mandible or maxilla is removed. Surgical excision of early-stage oral cancer can be performed easily with minimum loss of soft tissue and minor functional disability [19]. Significant removal of the tongue, floor of the mouth, or cheek lining can affect the oral anatomy enough to compromise the stability of a dental prosthesis. It is necessary to undertake appropriate reconstructive measures to restore soft-tissue volume and lining in cases where mucosal or soft-tissue volume loss has occurred [19]. This will help restore the mobility of the tongue and aid in mastication and swallowing.

Mandible resection is of two types: marginal mandibulectomy and segmental mandibulectomy. Marginal mandibulectomy performed in the premolar and molar regions of the mandible can lead to the flattening of the alveolar crest and the loss of mandibular sulci [20]. This can make it difficult for patients to use a removable dental prosthesis, which can become unstable. As a result, these patients may not be able to chew or swallow food satisfactorily. Furthermore, the feasibility of using dental implants in this situation is low due to insufficient bone height above the mandibular canal to allow for endosseous implants. A minimum distance of 1.5 mm is required between the inferior alveolar canal and the implant to prevent neuropathy of the inferior alveolar nerve [21]. Thus, sufficient residual bone is unlikely to be available in the posterior part of the mandible or the posterior part of the maxillary alveolus due to the immediacy of the maxillary sinuses. Marginal mandibulectomy performed in the anterior segment of the mandible between the mental foramina leaves adequate vertical height, enabling endosseous implants for a permanent fixed denture. For posteriorly located mandible lesions, performing a segmental mandibulectomy with fibula-free flap reconstruction is recommended instead of a marginal mandibulectomy. This allows for the consideration of endosseous implant placement [22].

Radiotherapy: The optimal delivery target, radiation type, and dose are crucial factors when considering a patient for dental rehabilitation. Ionizing radiation damages blood vessels in bone and adjacent tissues, leading to irreversible bone damage, tissue hypoxia, and a significant risk of osteoradionecrosis (ORN) following trauma or infection [23]. The risk of developing ORN is higher when performing surgery or dental procedures on irradiated bone due to compromised natural tissue repair processes [24]. The risk of ORN depends on the extent, progress, and irreversibility of ischemic alterations in the bone that are linked to the amount and timing of radiation therapy. Radiation doses above 40 Gy raise the likelihood of tooth decay and cavities, while a dose exceeding 60 Gy is significantly linked to the risk of ORN [24]. To ensure the accurate delivery of radiation to the targeted area, it is necessary to review the total dose delivery to the primary target and organ at risk (OAR) volume like the mandible, spinal cord, and larynx. Assessing the cumulative dose volume histogram and isodose curves on the treatment plan is necessary before the start of radiation treatment. This review can help determine the amount of radiation that may have been delivered to the surrounding healthy tissue due to the scatter effect. The information obtained from such a review can also help plan implant procedures. It is generally considered safe to perform dental interventions in areas of the mandible that are beyond the radiation ports.

Modern radiation delivery methods like IMRT can deliver accurate doses to the target area while minimizing exposure to surrounding healthy tissues [25]. However, when it comes to assessing the dosage of the tooth-bearing bone next to the target area, it becomes a complex process that requires the involvement of a radiation oncologist and physicist. Tumors located in the oropharynx, the lower gingiva, and the floor of the mouth in the oral cavity are associated with the highest radiation doses being delivered to the contralateral molar region [23,24].

Proton beam radiation therapy can help to reduce radiation exposure to adjacent normal tissues as its penumbra is much sharper and smaller than photons with minimal exit dose [26]. However, some radiation exposure to adjacent tissues is still unavoidable. In contrast, brachytherapy or administration of radioactive iodine may be considered safe for implant placement since high-dose exposure to the adjacent mandible is avoided, depending on the location of the primary tumor [27].

Chemotherapy: Cytotoxic chemotherapy can cause bone marrow suppression, resulting in an increased risk of bleeding during oral surgery [28]. Therefore, prior to any dentoalveolar surgery, patients undergoing chemotherapy should undergo screening to assess bleeding risk.

Post-treatment oral rehabilitative measures

Implant

Restoring complex maxillofacial defects presents a significant challenge and successful outcomes primarily depend on the precise replacement of the missing or deficient tissue. This complexity arises due to the unique anatomy of the affected area, individual patient expectations, and the distinctive nature of each defect. In the last two decades, personalized patient implants (PSIs) have made significant advances, thanks to the developments in computer-aided design and computer-aided manufacturing (CAD-CAM) technologies, additive manufacturing, and 3D printing [29]. PSIs offer a customized approach to reconstructive and esthetic surgery of intricate post-traumatic maxillofacial defects, as they are tailored to fit the defect with high accuracy. Furthermore, patient-specific reconstruction plates can be combined with bone grafts to restore contour following tumor resection, osteotomies, bone distraction, and grafting to enhance facial contour [29]. PSIs also suit onlay and inlay-type maxillary ridge augmentation and sinus lift procedures [30]. In situations where the deformity affects only one side of the face, the implant can be fabricated using a mirror image of the usual side, ensuring a more natural and symmetrical outcome.

Imaging and technology: Advanced medical imaging techniques such as computed tomography (CT) or magnetic resonance imaging (MRI) are employed to scan the area of interest before surgical procedures [29]. This step is crucial in creating PSIs and custom cutting guides utilized during surgery to execute precise bone cuts essential for tumor resection and reconstruction. These imaging modalities function primarily with two types of technologies, namely additive and subtractive manufacturing technologies, which are instrumental in the fabrication of PSIs [31]. 

Maxillofacial prosthesis materials: A maxillofacial prosthesis is a medical device used to replace or augment the hard and soft tissues of the head and neck region [32]. The ideal material for a maxillofacial prosthesis must satisfy specific criteria, such as being inexpensive, durable, radiolucent, lightweight, and biocompatible. Metals and polymers, particularly silicone, are commonly used to manufacture these prostheses [32]. Silicone, a polymerized dimethyl siloxane, is one of the earliest and most extensively used implant materials. It is used for soft-tissue augmentation and can be easily modified during surgery. Implant materials are broadly classified into two categories: absorbable and non-absorbable. Absorbable materials include poly-DL-lactic acid (PDLLA), polylactide-co-glycolide acid (PLGA), and calcium phosphate. Non-absorbable materials, such as hydroxyapatite (HA), are also used to fabricate maxillofacial prostheses. Polymethylmethacrylate (PMMA), polyether ether ketone (PEEK), ceramic, gold, cobalt-chromium (Co-Cr), zirconia, titanium, acrylic resin, and amorphous magnesium phosphate (AMP) blended with PEEK are also attractive options for maximum prostheses due to their high-temperature stability, wear, chemical and fatigue resistance, lightweight, high yield strength, durability, and biocompatibility. They do not cause any toxic or mutagenic effects and are resistant to attack by all substances except for concentrated sulfuric acid [32].

Zygomatic implants for asymmetrical maxilla: Zygomatic implants are a viable solution for patients who lack sufficient bone due to maxillary resection or atrophy. Placing these implants and the subsequent prosthetic treatment can be challenging due to the scarcity of supporting bone, soft tissues, and muscles. Several techniques have addressed this issue: tilted, pterygoid, short, wide, mini implants, various grafts, grafting the maxillary floor, and zygoma implants. Zygomatic implants, in particular, offer a non-graft alternative for severely atrophic maxilla and were first developed and reported by Brånemark [33]. The success rate of zygomatic implants is impressive, with an overall success rate of 97%. These implants are more extended and anchored in the zygoma, which provides excellent anchorage since the quality of bone is superior to that of the posterior maxilla, and the bone has broader and thicker trabecular bones that offer the initial primary stability required for loading [34,35]. 

Evaluation process: The evaluation process for zygomatic implants should include determining the appropriate incisal edge position, the need for lip support, and the appropriate vertical dimension of occlusion. These implants are usually inserted from the palatal side, are 30-50 mm long, and 4 mm wide, with a 45-degree tilted platform. They derive their anchorage in the zygoma and extend into the zygomatic process for good primary stability. They lie below the mucosa of the lateral wall of the sinus [34]. 

Implant placement protocols: Extreme care must be taken during the placement of these implants to obtain promising results since it is a technique-sensitive procedure. The maxilla must remain load-free for six months for healing before loading the prosthesis to allow the grafted bone to consolidate and the implants to be osseointegrated. It should not be considered the first line of treatment and is indicated in very few situations. In the prosthetic phase, the prosthesis should enable proper oral hygiene in the area and maintain adequate hygiene measures and mechanical demands related to the occlusion [33,34]. 

Initially, a two-stage procedure was recommended, but the original protocol has been replaced with immediate loading over time. Any adverse effects from immediate/early loading per se should be seen soon after commencing loading and not after a prolonged period. Provisional and final prostheses are developed to have a screw-retained structure that can be easily removed in the event of complications [34,35].

Drawbacks of zygomatic implants: The zygomatic implant technique application may result in restricted access to the surgical site and speech difficulties due to palatal implants. Additionally, the implant head may reduce the available space for the tongue. There is a risk of infection, overloading, hygiene maintenance issues, apical excess emergence in the infratemporal fossa, buccolingual fistula secondary to defective surgical closure, chronic gingivitis, severe sinus infection, and neurological impairment due to sinus perforation or intracerebral penetration of a zygomatic implant inserted in the pterygoid region if the surgical procedure is not performed precisely. The implant's placement at the pterygoid region requires careful attention. As such, it is paramount to evaluate the pros and cons of the zygomatic implant technique before proceeding with the procedure [34].

Classification of the maxilla for available bone: To determine the location of available bone sites in atrophic maxilla zygomatic implants, the Bedrossian classification of the maxilla is used [34]. This classification system is used as a reference for identifying zones and to guide the surgical approach for implant placement. This is done by reviewing the patient's panoramic radiograph. The maxilla is divided into different zones from I to IV (Table 1).

**Table 1:** Bedrossian classification

Zone	Anatomical location of available bone
I	Between canine to canine
II	Bicuspids
III	Molars
IV	Zygoma

The presence or absence of bone in these zones determines the surgical approach to be adapted. Without augmentation, treatment would follow guidelines as described in the subsequent table (Table 2) [34].

**Table 2:** Treatment guideline as per available bone in the maxilla

Available bone in various zones	Treatment plan
Adequate volume of bone in zones I, II, and III	To put 4-8 implants in an axial (non-angled) direction
Inadequate bone in zone III and sufficient volume in zones I and II	ALL-on-X approach would be followed with 4-6 implants placed axially or angled (tilted) to achieve the widest arch spread for a better A ratio
Adequate bone only in zones I and IV and insufficient volume in zones II and III	Put 2-4 implants in zone I and a zygomatic implant bilaterally in zone IV
Inadequate bone in zones I and II	To be treated with dual bilateral (quad) zygomatic implants
Zygomatic implants have platforms with inadequate spread, but sufficient bone is present in zone III	Pterygoid implants may be added to increase the A-P spread to allow restoration of the arch

Suggested criteria for the success of zygoma implants: A slight degree of mobility may manifest during the evaluation of individual zygomatic implants, although without any other pathological indications. This mobility emerges due to the flexible modulus of the anchoring zygomatic bone when subjected to a remotely applied force, which causes it to flex. If the movement is not rotational and dissipates, it is not a cause for concern. However, if there is a rotational movement, it should be deemed an indication of implant failure. To prevent implant failure, implants are commonly connected. It is also essential to be aware of peri-implantitis, which can lead to implant failure [34,35].

Major and minor criteria can be used to diagnose associated sinus pathology (Table 3). The diagnosis requires two or more major criteria, one major and two or more minor criteria, or purulence on nasal examination [35]. 

**Table 3:** Major and minor criteria to diagnose associated sinus pathology

Major criteria	Minor criteria
Facial pain or pressure	Headache
Facial congestion or fullness	Nonacute fever
Nasal obstruction	Halitosis
Purulent discharge	Fatigue
Hyposmia or anosmia	Dental pain
Acute fever	Cough
-	Otalgia or aural fullness

Obturators in Postsurgical Cancer Patients

An obturator is a prosthesis used to close palatal defects caused by congenital or acquired conditions. Maxillectomy defects can lead to oroantral communication, making eating, speaking, and swallowing difficult and causing facial disfigurement and drooling. In cases where significant defects cannot be surgically closed, prosthetic obturators are the best treatment option. Although surgical procedures like microvascular free flaps can also close minor defects, they are often associated with increased hospitalization and high morbidity in the flap donor area [36].

History: As early as the 1500s, Ambroise was the first person known to use an artificial device to close a palatal defect. Later, in 1875, Claude Martin described using a surgical obturator prosthesis, while Fry described using impressions before surgery in 1927. Steadman then described using an acrylic resin prosthesis lined with gutta-percha in 1956 to hold a skin graft within a maxillectomy defect [37-39].

Classification of defect: The extent of the defect and preservation of dentition and bone is described in Table 4 [40].

**Table 4:** Classification of the extent of the defect and preservation of dentition and bone

Class	Extent of the defect
I	Defects involve the removal of the dentition and alveolar bone in the midline
II	Defects preserve the premaxilla on the defect side
III	Defects are located in the center of the palate, but dentition is maintained, which is the most advantageous situation as teeth provide retention to the obturator
IV	Defects contain the premaxilla on the side opposite to the surgery
V	Defects maintain the anterior teeth, but the posterior teeth, hard palate, and a varying part of the soft palate have been removed
VI	Defects affect the premaxilla and are usually driven by accidental trauma followed by surgical removal

Types of obturator: The Keyf protocol is a widely used construction protocol in surgeries. It involves three stages - surgical, interim, and definitive. The first stage, known as the immediate or surgical obturator phase, creates a base plate appliance using the preoperative impression cast. This appliance is inserted during maxilla resection or removal surgery and is secured using wires or tiny screws. The base plate acts as a matrix for the surgical packing, ensuring the close adaptation of the skin graft. This, in turn, reduces the risk of oral contamination and local wound infection [37].

After maxillofacial surgery, patients may require an obturator prosthesis, which is an interim device that fills the gap created during the surgical procedure. It prevents irritation of non-cicatrized and bleeding tissues, minimizes facial disfigurement and scar contraction, restores speech and deglutition functions, and provides psychological benefits to the patient by helping them regain social acceptance. The surrounding tissues may undergo changes, such as reorganizing scar tissue, which can cause the interim device to require frequent adjustments until the tissues stabilize. Once stabilization occurs, a permanent device can be created. If the gap is significant, additional support may be necessary to prevent the obturator device from rotating. The transitional or interim obturator serves as a link between the surgical and final stages of treatment. It is usually provided 7-14 days after surgery and worn until the surgical site is fully healed and has stabilized in size. During this period, the transitional prosthesis is inserted and adjusted until the healing is complete, which can take two to 24 months. It aids in maintaining oral hygiene [36-41].

Various types of obturators are available to address the unique needs of patients, including solid bulb obturators, open and closed hollow obturators, inflatable obturators, and two-piece hollow obturator prostheses. In cases where deep undercuts are present, soft liners may be utilized. However, interim obturator prostheses should not include posterior teeth, as doing so could significantly increase stress on the wound and delay the healing process [41].

The temporary obturator is created using an impression cast that includes an artificial palate and ridge but omits teeth. To maintain cleanliness, it is recommended that old lining material be removed entirely to avoid possible bacterial contamination through porosity. Replacement of lining material should occur periodically to ensure sanitary conditions. However, interim lining material may increase the size and weight of the obturator, and the temporary material may become unhygienic and rough [37].

Obturator designing criteria: The final obturator, designated for long-term use, is created from a maxillary cast obtained after healing. Fixed prostheses (crowns) and/or removable prostheses are constructed only after healing. It is important to note that changes associated with healing and remodeling will continue to occur in the border areas of the defect for at least one year. These dimensional changes primarily relate to the peripheral soft tissues rather than the bony support areas [36,37,41-44].

When designing an obturator prosthesis, it is crucial to follow basic prosthodontic principles. These principles include distributing stress over a wide area, using a sturdy major connector to stabilize the prosthesis, and placing retaining components within the arch to minimize functional forces that could cause dislodgement. It is important to ensure that the occlusion force is evenly distributed in both centric and eccentric jaw positions to minimize the movement of the prosthesis. Lateral forces can generate stress, which can be minimized by selecting an appropriate occlusal scheme, eliminating premature occlusal contacts, and using a wide distribution of stabilizing components [37,41].

Factors affecting retention of obturator: The retention of an obturator prosthesis depends heavily on the remaining teeth. The number, position, and health of the remaining teeth are important factors in determining how much stress the remaining teeth can handle. If the remaining teeth are situated on one side, an intracoronal retainer might help reduce the vertical movement of the prosthesis within the defect. Intracoronal retainers might be used for minor defects and stable remaining teeth. However, if the defect is significant or some or all of the remaining teeth are weak, extracorporeal retainers should be used. Guiding planes can be added to prevent the obturator from vertically shifting and to keep the retentive clasp arms from disengaging if the remaining teeth aren't parallel to the defect's walls or if the palatal surfaces of the teeth are improper. Using acrylic resin teeth with a smaller occlusal contact area is recommended. Changing the cusp angle of posterior teeth affects the prosthesis's stability on an edentulous resected maxilla [37,40].

In some instances, it may become necessary to accommodate an occlusal imbalance of the edentulous mandible or maxilla in eccentric occluding positions. In such instances, non-anatomical posterior teeth are preferred. These teeth are positioned in centric relation and adjusted to eliminate lateral deflective occlusal contact. The tissue in the area changes for at least one year due to scar contracture and further wound organization. As a result, the prosthesis must be rebased to account for these changes. Since the tissues that support a maxillofacial prosthesis can change rapidly, occlusion and base adaptation must be frequently re-evaluated and corrected through selective grinding of the occlusion or rebasing of the prosthesis. In patients with remaining teeth and soft-tissue undercut, retention, support, and stability are critical for the obturator. Retentive aids like magnets, snap-on (friction-type) attachments, acrylic buttons, retentive clips, and implants are employed to achieve this goal. The use of implants is a recent advancement in maxillofacial prosthodontics, which effectively enhances the retention of prostheses without the need for other appliances [37,40,41,45].

It is essential to ensure that the significant connectors in dental prosthetics are rigid and that occlusal rests guide occlusal forces along the long axis of the teeth. Guide planes should be designed to promote stability and bracing, while retention should be within the physiological limits of the periodontal ligament. It is also essential to gain maximum support from the residual soft tissues. The metal framework obturator prosthesis is a durable material with high thermal conductivity, which makes it sensitive to temperature changes [40].

For dentate patients, conventional obturator materials are chromium-cobalt frameworks with PMMA, while edentulous patients only use PMMA. Dental or zygoma implants to support obturator prostheses can improve masticatory function. Both patients and healthcare providers must know the proper care for obturators. After meals and before bedtime, the obturator should be cleaned with soap and water or a dental brush and paste to remove plaque. Commercial denture-soaking agents can be used to remove stains. The prosthesis should be placed in a water container at night and moved around. It is important to note that surgical obturators should not be removed [37,46].

Flexible Dentures

Maxillectomy patients require prosthetic rehabilitation to replace missing oral and facial structures and to restore functions like eating, speaking, and swallowing. Prostheses support the orbital contents, prevent enophthalmos and diplopia, and restore midfacial contour. Patients may experience discomfort in their oral tissues due to reduced saliva flow, resulting in issues with denture retention and decreased bactericidal action [47].

In maxillectomy patients, the rehabilitation is done by maxillofacial prosthesis. Usually, PMMA is used as a definitive prosthesis, but flexible dentures are an excellent alternative to conventionally used dentures. One of the main advantages is that they do not cause sore spots due to negative reactions to acrylic resins [48]. They are made from a flexible material that absorbs small amounts of water, which makes the denture soft and tissue-compatible while also being nearly unbreakable. The material used is durable, heat-resistant, and ductile. They are designed using a softer material that can lock into the undercuts of the ridges. The clasps are built to curl around the neck of teeth, blending in with the natural gums, adapting to the constant movement and flexibility in the mouth, and providing stability for eating tough foods. They can be fabricated quite thin, disengage forces on individual teeth, preventing the transfer of forces to the remaining natural teeth and the other side of the arch, light in weight, heat-resistant, and ductile. It is injected at a temperature of 274° to 300°C, ensuring the durability and longevity of the prosthesis, even when subjected to high temperatures [49].

Flexible dentures are a great option for those looking for excellent esthetics, as the material's translucency picks up underlying tissue tones, making them almost impossible to detect in the mouth. They are also more comfortable and stain-resistant, and there are no visible clasps on tooth surfaces when clear clasps are used in manufacturing [50]. They are biocompatible and do not cause allergic reactions, as the material is monomer-free. They are also less likely to warp or become brittle, as they are fabricated using the injection-molded technique. They are more comfortable and hygienic because the material used is non-porous, so bacteria build-up does not occur within them, unlike acrylic dentures, where more colonization has been found on the tissue surface of the maxillary denture. They are also quicker to construct than hard acrylic dentures, with the trial fitting often being used as part of the final denture. In conclusion, flexible dentures are a highly desirable option due to their many advantages over traditional hard acrylic dentures [51-57].

**Figure 1:**
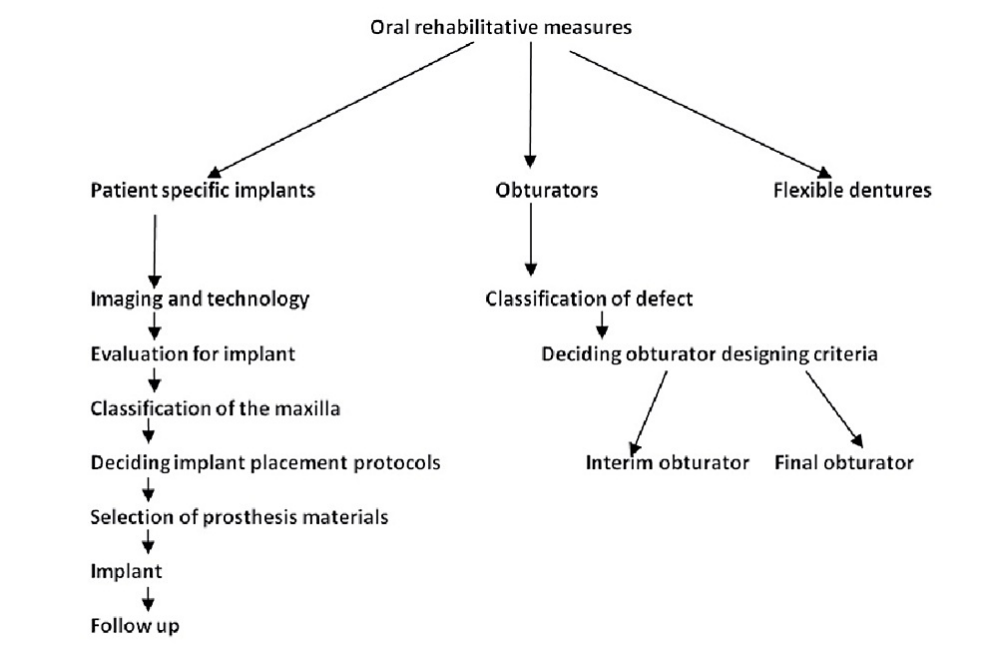
Flow diagram for dental rehabilitation options

The flow chart of dental rehabilitation measures is depicted in Figure 1. 

Some common peri- and post-treatment complications and specific rehabilitation measures are discussed hereon. 

Challenges in maintaining oral hygiene

Managing oral hygiene in patients with HNC is a complicated process that requires specialized care and support. The objectives of this management may differ based on the type of cancer treatment administered. Therefore, the role of a dental hygienist is critical in screening for oral health issues and managing oro-dental complications resulting from surgery, radiation therapy, and/or chemotherapy. The dental hygienist significantly promotes personal care to enhance the patient's QoL. They help patients achieve good oral hygiene, remove tartar deposits that can lead to infection, maintain healthy oral mucosa, and educate patients on how to handle treatment-related issues and equip them with the necessary tools for prevention [58].

A maintenance program and regular professional oral hygiene follow-ups are crucial for the patient's long-term oral health. The maintenance program involves encouraging and instructing patients on oral hygiene, conducting routine oral mucosal health check-ups, and providing professional oral hygiene services every three months. In summary, managing oral hygiene in patients with HNC requires a collaborative approach between dental hygienists, oncologists, and other healthcare professionals [58]. This approach ensures patients receive the specialized care and support necessary to maintain their oral health and well-being.

Radiation-Induced Mucositis

Radiation-induced oral mucositis is a condition where there is rapid atrophy and erosive lesions in the oral mucosa due to direct and indirect radiation damage. This condition causes pain, dysphagia, and feeding difficulties and can also lead to fungal overinfection, which exacerbates the pain and difficulty in swallowing and feeding. When radiotherapy is done to the head-neck region, salivary flow decreases, resulting in xerostomia and dysphagia [58,59]. Therefore, any periodontal causal therapy and dental prophylaxis should be initiated at least 15 days before the commencement of radiation therapy to reduce the risk of ORN and other biological complications [59].

Management options: Various studies have evaluated the efficacy of various types of mouthwashes in managing oral mucositis during radiotherapy, although none of them was recommended as category 1 evidence by international guidelines. Huang et al. reported that using a saline-based mouthwash during radiotherapy can enhance the QoL without affecting symptomatology or the onset of mucositis [60]. Manifar S, et al. conducted a double-blind, randomized controlled trial that demonstrated a significant reduction in the intensity of oral mucositis with a probiotic mouthwash [61]. Sio et al. found that using doxepin or diphenhydramine-lidocaine-antacid mouthwash is ineffective in preventing mucositis, but they can alleviate the pain associated with this condition [62]. Moreover, the multicenter study conducted by Lalla et al. revealed that a mouthwash made of hydrogen peroxide, eugenol, camphor, and parchlorophenol can prevent radiation-induced mucositis [63].

Studies have also compared the effectiveness of plant extract and natural substance-based mouthwashes with saline, baking soda-based, or placebo mouthwashes. The effectiveness of these mouthwashes is often comparable to bicarbonate-based saline solution rinses [64-66]. Granulocyte-macrophage colony-stimulating factor mouthwashes are the most studied for the prevention of mucositis [67]. Antiseptic mouthwashes alone can help improve oral hygiene and prevent overinfections [68].

Low-level laser therapy is also used to manage oral mucositis. It involves exposing the affected parts of the mucosa to a 15-minute laser treatment, during which 180 Joules are delivered. The therapy focuses on the areas with the most inflammation or mucositis lesions [69].

Prevention of Postoperative Infection 

Patients who undergo oncosurgery are at an elevated risk of developing postoperative infections, including pneumonia. Oral care programs are recommended for both pre- and post-surgery to mitigate this risk. Recent research conducted by Ishimaru et al. has demonstrated that preparing patients with oral prevention pathways significantly decreases postoperative pneumonia and all-cause 30-day mortality following cancer resection [70].

During the post-surgical phase, patients should adhere to a regimen of proper oral hygiene practices at home. This includes utilizing a small-headed, soft-bristled toothbrush, a gentle brushing technique, fluoride toothpaste, and applying topical fluoride gel once a day for 5 minutes (twice a day during radiotherapy). Additionally, patients should use alcohol-free 0.12% chlorhexidine rinses and saline solution rinses, engage in interdental hygiene with dental floss or an interdental brush, and perform tongue cleansing with a soft toothbrush or gauze [70].

For patients with removable dentures or palatal obturators, it is essential to brush prosthetic restorations with a denture brush and Marseille soap and then soak them daily in a solution with water and 2% sodium hypochlorite or chlorhexidine to disinfect them [71]. By following these straightforward steps, patients can significantly reduce the risk of postoperative infections and expedite their recovery from surgery.

Radiation-Induced Caries Prevention

Radiation-induced caries is a common side effect of radiotherapy, which usually occurs about three months after treatment. These caries are often found at the dental neck level and are caused by a decrease in salivary flow, changes in saliva quality, and a decrease in pH levels. To prevent this, it is recommended to maintain excellent oral hygiene and use topical fluoroprophylaxis. Research has shown that using biomimetic CPP-ACP saliva and SnF2/NaF significantly reduces caries progression in patients receiving radiotherapy in the head-neck region [72]. Additionally, preventive fluoride applications have positively impacted the oral health of radiation patients in the head-neck region, decreasing caries, plaque index, and gingival inflammation [72].

Speech and deglutition impairments 

Speech impairments are a common problem after surgical treatment involving changes to the oral anatomy. This can significantly affect the QoL of patients. The severity of speech problems can be influenced by various factors, such as the patient's age, cancer stage, site of origin, treatment approach, and degree of resection [73]. The success of rehabilitation after reconstructive surgery depends mainly on the quality of the surgery and the rehabilitation process. While preserving the organ's anatomy is preferable, it does not always guarantee preserving its function. Even when treatments allow for organ preservation, such as laser or robotic surgery or radiation therapy in combination with chemotherapy, they can still result in significant functional complications [73,74].

Speech Therapy

Speech therapy plays a crucial role in speech rehabilitation. It is recommended to start as soon as cancer is diagnosed, even before treatment. Building a connection with the patient and their family is important for better adherence to treatment and benefits. However, in most cases, speech therapists are only involved in the healing phase and rarely part of a multidisciplinary team during the pre-treatment phase and therapeutic decision-making [75-77].

During the evaluation process of HNC patients, speech therapists consider various factors such as oral motricity, voice, swallowing ability, articulation pattern, the presence of feeding tubes, provisional or permanent tracheostomy, and respiratory conditions. During initial treatment, speech therapy focuses on rehabilitating swallowing. Patients who undergo surgery may require alternative feeding routes, which are removed once the patient can safely swallow. The speech therapist then reintroduces the oral route and determines the appropriate food consistency through training involving various exercises like sensory stimulation, airway protection maneuvers, postures, and strengthening exercises [75-77].

The process of vocal rehabilitation for patients who have undergone total laryngectomy is quite complicated as the surgery directly affects their voice box, causing a complete loss of vocal ability. To help these patients regain their ability to communicate, speech therapists should provide guidance and support before the surgery by introducing various methods for vocal rehabilitation, educating them about stoma care after the surgery, and helping them choose the appropriate supplies [78,79].

Palatal Augmentation Prosthesis

The palatal augmentation prosthesis (PAP) is an effective solution for speech problems that are not resolved through reconstructive surgery and speech therapy. A recent meta-analysis examining the effectiveness of PAP on speech problems showed that it successfully improved pronunciation, especially with consonants. Despite its effectiveness, there are few reports on PAP morphology. Therefore, palatograms are required to determine PAP morphology based on the resected tongue morphology and reconstructed flap morphology [80-82].

Xerostomia

Xerostomia, or oral dryness, is when the mouth feels drier than usual. This condition often occurs during oral cancer treatment due to the effects of surgery, radiation, and chemotherapy on the salivary glands. These treatments can cause organic changes in the salivary gland tissue, leading to xerostomia [83,84]. Approximately 80% of patients who undergo radiation therapy to the head and neck experience some degree of xerostomia. During the first week of radiation therapy, there is a significant decrease in salivary flow by 50% to 60%. As conventional radiation therapy continues for seven weeks, the salivary flow of the affected glands reduces to approximately 20%. After undergoing radiation therapy for HNC, the salivary function may continue to decline for a few months. While some degree of regeneration in saliva production may occur, many survivors still face the challenge of chronic dry mouth. This decreased saliva production can worsen the QoL for survivors by reducing their ability to taste food and making it uncomfortable or even painful to chew it. Additionally, decreased saliva production can increase the risk of dental caries and mucosal fissures [83-86].

Prevention of Xerostomia

Preventing radiation-associated xerostomia is the best method for reducing its impact on a patient's QoL after oncologic therapy. Xerostomia is a predictable and non-random effect of radiation exposure. A threshold exists for radiotherapy doses below which damage to relevant salivary glands can be reduced or prevented. In recent years, clinically validated dose constraints have been established for salivary-associated OARs, such as parotid glands, submandibular glands, and oral cavities. With the introduction of IMRT, oncologists could use an inverse planning algorithm and multiple fields to spare OARs from toxic threshold doses [87,88].

Management

Two common approaches to alleviating xerostomia in patients are salivary substitutes and oral/peripheral stimulation. Salivary substitutes are widely available commercially without a prescription for cancer and non-cancer patients with xerostomia. Unfortunately, these are notorious for providing short-lived relief to patients. Numerous studies have focused on different saliva substitutes and compared their effectiveness with water alone. However, many of these studies are flawed in design, lack objective clinical data, or are ambiguous in their recommendations. Some studies have found that drinking water frequently can be as effective as using saliva substitutes. Mucoadhesive discs of xylitol or sugar-free gum have been used with varying results [83-88].

Salivary substitutes cannot offer the same level of antimicrobial and immunologic functions as natural saliva. Therefore, additional products such as antimicrobial rinses and oral hygiene agents must be used to achieve similar levels of protection. Pilocarpine has been tested as a treatment for dry mouth syndrome, particularly in patients with Sjogren's or those who have undergone radiotherapy. It is available in the form of topical mouth rinse/spray, lozenges, or systemic tablets. Several studies have compared the effectiveness of these treatments to no treatment, placebo, or each other. However, these studies have not provided conclusive evidence of significant improvements in outcomes. Systemic administration of some therapies can cause side effects that may make some patients ineligible. For instance, patients with asthma, glaucoma, cardiovascular disease, or respiratory illnesses like chronic obstructive pulmonary disease may not qualify for such treatments [89,90].

Cevimeline is a type of parasympathomimetic drug that can help alleviate symptoms of Sjogren's syndrome. Some studies have also explored the use of cevimeline to treat dry mouth caused by radiation therapy, with some positive results. However, some patients experienced side effects such as sweating and digestive problems, which hindered their ability to continue taking the drug. Once again, contraindications to cevimeline may further challenge its implementation [91].

Acupuncture has gained popularity as a therapeutic intervention for xerostomia. Three studies have been carried out to evaluate its efficacy. One of these studies compared sham acupuncture to targeted acupuncture and failed to demonstrate significant improvement. However, another study revealed that acupuncture can alleviate dry mouth symptoms in some patients after radiation therapy by stimulating the residual salivary gland tissue. Blom et al. reported a noteworthy increase in unstimulated and stimulated salivary flow rates in individuals with xerostomia of different origins, including radiation therapy, after receiving 24 acupuncture treatments [92-94]. Further research is required to ascertain the comparative effectiveness and replicability of the outcomes [92-94].

Trismus 

Trismus, which is characterized by the inability to open the mouth fully, is a commonly observed oral disorder in patients receiving therapy for HNC and is the second most prevalent oral morbidity after xerostomia. The incidence of trismus is often linked to the combined effects of chemotherapy, surgery, and radiation therapy, with prevalence rates ranging from 5% to 42% [95].

Etiopathogenesis

Radiation therapy may lead to significant masseter, temporalis, and medial pterygoid muscle exposure, causing trismus. Radiation-related trismus is typically attributed to fibrosis, scar tissue formation, nerve damage, muscle atrophy, or a combination of these factors, leading to limited mobility of the muscles of mastication. This condition can result in progressive radiation fibrosis, which may develop five to 10 or more years after therapy [96]. Surgical removal of cancer through transoral laser or robotic resection may result in scarring, which in turn may cause trismus in the short and medium terms, leading to the possibility of permanent mouth-opening problems if aggressive therapy is not initiated [97]. Therefore, clinicians should be aware of the potential risk of trismus and carefully monitor patients who have undergone therapy to ensure appropriate management of this condition. Since a majority of HNCs occur in the oral cavity or oropharynx, trismus is likely to become an increasingly common and devastating sequela of treatment.

Trismus has significant physical, psychosocial, and safety implications. This condition affects vital functions such as swallowing, communication, and oral access, leading to food insertion, biting, chewing, speech, and dental examination difficulties. Individuals with trismus are also at an increased risk of aspiration, oral intubation, poor oral hygiene, and emesis [95-98]. A recent survey conducted on survivors of oropharyngeal cancer revealed that patients who suffered from trismus had a decreased QoL and a negative association between diet and the severity of trismus [99].

Psychologically, trismus can lead to fear or embarrassment in patients, which may cause them to avoid eating in public, intensifying social isolation and increasing the risk of depression. In extreme situations, patients may even experience increased suicidal tendencies. Furthermore, trismus can exacerbate the difficulties of communication, leading to alienation and isolation. In conclusion, trismus is a debilitating condition that has numerous physical, psychosocial, and safety implications [99,100].

Conservative Management

Implementing conservative treatment methods and providing relevant instructions are vital in preventing severe trismus in patients. Early exercise therapy initiation plays a crucial role in this context. Early manual and mechanical opening training is essential for successfully preventing trismus. An investigation through a randomized controlled trial revealed significant differences in mouth opening at three to six months for patients with HNC who underwent prophylactic swallowing exercises that included mouth-opening training [101]. These findings demonstrate the importance of early initiation of exercise therapy, mainly manual and mechanical opening training, in preventing trismus successfully. Patients need to receive conservative treatment methods and instructions to prevent severe trismus. According to a systematic review, a significant variation exists in the treatment programs employed for trismus, including different stretching devices, techniques, duration, and repetition. Although most therapeutic exercise programs for trismus are reactive and demonstrate some improvement, they fail to restore the maximum interincisal opening (MIO) to a normative range [101,102]. Currently, several tools are available for stretching, but a systematic review conducted by Kamstra et al. could not determine a preferred exercise therapy [102].

Manual therapy is a comprehensive term that refers to a range of soft-tissue mobilization methods that utilize physical and movement-related manipulations of the body. This approach encompasses a variety of techniques, including manual lymphatic drainage, myofascial release, massage, and passive and active stretching. These techniques apply variable degrees of pressure and stretch connective tissue and joints to restore fluid transport and range of motion. Manual therapy techniques effectively improve circulation, reduce local ischemia, stimulate proprioception, decrease muscle spasms and adhesions, and enhance MIO in patients suffering from temporomandibular joint disorders [103]. Generally, manual therapy is considered a safe intervention, although it can potentially cause harm to hard or soft tissues. Most cases show a loss in MIO between the perioperative period and the end of follow-up, indicating the expected degree of trismus refractoriness. However, immediate physical therapy and the use of a mouth-exercising device may aid in preserving the MIO. The role of pentoxifylline in managing trismus is yet to be clarified, as only one pilot study has evaluated the efficacy of pentoxifylline in increasing mouth opening [104].

Surgical Management

In some instances, conservative therapies may fail to achieve an adequate degree of mouth opening necessary for essential daily activities. In such situations, surgical intervention is a viable option. Despite the high prevalence of trismus and its impact on the QoL of patients with HNSCC, no clear therapeutic flowchart for the surgical release of trismus is currently available. Surgical release strategies for trismus may comprise one or a combination of several approaches, including a myotomy of the masticatory muscles, coronoidectomy, and resection of fibrous scar tissue followed by free flap reconstruction [97,105].

An analysis revealed that scar tissue release using Functional Force Release (FFR) was significantly less effective in achieving an MIO increase than the coronoidectomy group. However, comparing the groups is prone to bias, as the preoperative MIO was lower in the population where an FFR was performed. Although a myotomy is considered one of the most accessible methods for trismus release, none of the original research teams conducted a myotomy without an FFR or coronoidectomy. The consensus in the literature is that a myotomy is insufficient in releasing the MIO [97,105]. One of the primary reasons for this is that the installed fibrous tissue is the leading factor contributing to chronic trismus, especially for severe cases of trismus. Due to the multifactorial nature of this complication, the three described release methods are often combined, making it difficult to distinguish the methods' separate effects on MIO. Further research is indispensable to a larger homogeneous population, allowing a better understanding and comparison of the surgical options.

Recurrent Trismus

The recurrence of trismus after myotomy is a common outcome owing to the reformation of fibrosis. Silberstein et al. have identified a possible role of botulinum toxin A in the myotomy procedure. According to their study, administering botulinum toxin A into a muscle immediately after myotomy could disrupt muscle healing and improve the long-term outcome. It is observed that higher gains in MIO are expected after trismus release when no primary resection was performed [106]. This can be attributed to the formation of fibrous scar tissue following primary surgery.

Gustatory dysfunction

Gustatory dysfunction or dysgeusia is a common condition that affects taste and can manifest in various forms during HNC treatments. It is often an adverse effect of radiotherapy and can lead to malnutrition and a significant impact on a patient's daily QoL. Gustatory dysfunction is often overlooked compared to other adverse effects of radiation therapy. 

During radiation therapy, taste progenitor cells may undergo cell cycle arrest and apoptosis, resulting in changes to taste receptors' sensitivity, chemical interaction with taste substances, and diffusion of taste substances to the taste receptors [107]. Gustatory dysfunction typically begins during the first week of radiation treatment and intensifies in the second week, reaching its peak around the third to the fifth week. The temporary loss of taste is common but usually recovers in a year. In some cases, it may take up to seven years to fully recover. Patients who receive radiation doses exceeding 60 Gy are likely to experience significant gustatory dysfunction. Other factors, such as oral mucositis and salivary hypofunction, can increase the likelihood of gustatory impairment during radiation therapy [108].

Patients with cancer in the oral cavity and oropharynx are more likely to experience severe taste impairment than those with cancer of the nasopharynx, hypopharynx, or larynx because of the damage inflicted on normal tissues in the radiation portals, particularly the volume of the tongue in the irradiated field. The proportion of the tongue within the treatment field is significantly associated with subjective and objective taste loss [107,108].

Management

There are few studies that have investigated the impact of radiation therapy on the four basic taste qualities: sweet, salt, sour, and bitter. Most studies have found that bitter and salt qualities are affected earliest and to the greatest extent [108,109]. The recovery of gustatory dysfunction following treatment is highly variable [108,109]. To help improve dysgeusia, healthcare professionals can lower the oral cavity radiation dose and offer pre-counseling for gustatory dysfunction and a modified diet [108-111]. Educating patients about gustatory dysfunction and its recovery pattern is crucial, enabling them to manage treatment-related side effects effectively. Recent research has shown that umami, the fifth basic taste, decreases during the third week after radiotherapy but typically recovers as early as the eighth week [112]. Therefore, an intervention method effectively utilizing umami is expected to be helpful. Although no other intervention studies have demonstrated efficacy in improving taste loss, adopting culturally sensitive compensatory methods is advisable. Food culture significantly impacts taste loss, and culturally sensitive interventions can be more effective.

Neuropathy

Patients receiving radiotherapy for HNC have a higher risk of developing neuropathies due to the abundance of nervous tissues in the head and neck region [113]. Radiotherapy can cause long-lasting damage to the nervous system because nervous tissues have limited capacity for repair and regeneration. It is crucial to understand HNC radiotherapy-induced neuropathies because they are irreversible and can significantly impact patients' QoL.

Peripheral Neuropathy

A peripheral nerve radiation insult is defined by the formation of perineurium and altered axonal growth. On a cellular level, radiotherapy targets several molecular entities, including DNA integrity, oxidative stress, inflammatory cascades, and synaptic activity. Ultimately, this molecular damage leads to apoptosis, neuroinflammation, and altered neuronal function, the primary characteristics of radiation-induced neuropathies in HNC [114,115]. Chemotherapy drugs, such as cisplatin and taxanes, can also result in peripheral neuropathy. This disorder is referred to as chemotherapy-induced peripheral neuropathy (CIPN) [116]. Additionally, cancer itself may contribute to the development of CIPN by altering peripheral nerve function [117].

Sensorineural Hearing Loss

One of the common neurological complications that patients with HNC experience is sensorineural hearing loss (SNHL) after undergoing radiotherapy. The risk of developing SNHL is higher when the radiation field includes the inner cochlea near the primary tumor site. Damage to the stria vascularis and organ of Corti of the cochlea due to radiation is the leading cause of auditory impairment [118]. Moreover, patients receiving chemoradiotherapy are at an increased risk of developing SNHL, especially when treated with cisplatin, a well-known ototoxic agent [117,118].

Olfactory Dysfunction/Dysosmia

Dysosmia, characterized by a distortion of the sense of smell, can still significantly impact the survivors' daily lives. This includes their food intake, well-being, and ability to detect safety hazards [119]. Insufficient research has been conducted on olfactory dysfunctions caused by radiation in patients with HNC. This is mainly due to the infrequent occurrence of nasal cavities and paranasal malignancies, which only account for 3% of HNC cases. Additionally, the olfactory area is located in the upper part of the anatomy, which is typically unaffected by radiation treatment for the more common types of HNC [119]. Furthermore, olfactory neuroepithelium has a faster regenerative rate compared to other tissues.

A recent systematic review found that radiation therapy impaired odor detection and identification thresholds immediately after treatment. This effect was compounded if patients had concurrent chemotherapy. Although odor perception could recover as early as six months after treatment, olfactory recuperation could be prolonged up to 20 months if patients received a dose higher than 10 Gy to the olfactory epithelium [120]. In some cases, olfactory alterations were still reported up to five years after radiation therapy. Unfortunately, no radioprotective agents against dysosmia have been adopted yet. However, previous studies have shown that olfactory training can improve patients' sense of smell and could be helpful [121].

Radiation-induced neuropathy is a complex condition that can take on many forms based on factors such as the type of treatment, the location of the radiation, and the patient's overall health. Although current medical treatments can provide some relief for the associated side effects, they are often limited in their effectiveness. Additionally, preventive measures have only had partial success [113-121]. Therefore, further research is necessary to gain a better understanding of the mechanisms that cause neural injuries following radiotherapy for HNC and to develop more effective interventions.

The psychological impact of HNC treatment

HNC can cause significant limitations in daily activities, leading to psychosocial challenges that impact the patient's QoL. The psychological effects of HNC pose a significant challenge in terms of understanding and treating them. These mental health issues can have adverse effects on one's work, family life, and future plans. After treatment, cosmetic and functional impairments can reduce the chances of returning to work and significantly impact the patient's QoL.

Spectrum of Psychological Impacts

HNC patients commonly experience psychological symptoms such as anxiety, depression, and fatigue, especially after receiving radiotherapy [122]. The prevalence of depressive symptoms among HNC patients varies widely from 6% to 42%, depending on the duration since diagnosis [123]. Anxiety coupled with the apprehension of cancer recurrence is a common concern among cancer patients, and HNC patients may also experience anxiety related to loss of occupation, social isolation, and social status [122,123]. Fatigue is a common complaint among 18-25% of patients who have received therapy for HNC [124]. Functional impairments can also lead to esthetic changes and worsening social contact loss.

Furthermore, HNC survivors often experience post-traumatic distress, which has been well documented [125]. There is a 12 times higher rate of suicide among oral cancer patients than the general American population [126]. These effects can manifest as uncertainty regarding the likelihood of cancer recurrence, disruption of daily routines, feelings of diminished self-esteem, efforts to understand the changes taking place, and the search for a viable way forward. Fluctuations in mood can hinder treatment progress, while long-term psychological states can persist for months or even years after initial treatment [123-128].

Management

It is crucial to identify practical interventions that can manage the diverse needs of HNC patients and improve their overall QoL. It is unclear whether having a job helps to alleviate depressive symptoms or if individuals who are less prone to depression are more likely to return to work. Evaluating the QoL of patients with oral cancer is crucial in both clinical and research settings. Several questionnaires, such as UW-QOL, MDADI, and EORTC QLQ-H&N35, have been developed and used for this purpose [129-132]. It should be routine for a psychiatrist to assess and intervene in affected cases, but it needs to be utilized more.

Limitations of review

Please be advised that this paper covers the majority of complications associated with oral cancer treatment. Nevertheless, there may be rare complications that have not been addressed in this study, and some treatment options may have been omitted due to the difficulty of incorporating every piece of literature into a review paper. Furthermore, it should be noted that some treatment methods may have been less effective or have failed and, as a result, are not incorporated in this review. The articles cited in this paper were selected using the narrative review method, which may introduce some bias into the review. It is recommended that systematic reviews be conducted in the future to ensure a more comprehensive and impartial analysis of existing research in this field.

## Conclusions

Rehabilitating the patient's appearance, speech, dentition, and swallowing ability after HNC treatment is crucial. Unfortunately, there are very few established follow-up programs for post-treatment oral cancer patients in daily clinical practice. Some measures like PSIs, obturators, and flexible dentures offer a personalized approach to rehabilitation after maxillofacial surgery. Oral hygiene is paramount after HNC surgery and requires specialized care.

Radiation-induced caries, speech impairments, and xerostomia are common side effects of treatment that can be managed with proper care and therapy. HNC patients may experience trismus, gustatory dysfunction, neuropathies, and psychosocial challenges. Proper follow-up and management are crucial for preventing and improving QoL. Despite its importance in improving patients' QoL, rehabilitation is often overlooked. Our review covers factors affecting rehabilitation, providing practical insights to professionals to inspire positive changes.
